# Comprehensive Behavioral and Molecular Characterization of a New Knock-In Mouse Model of Huntington’s Disease: zQ175

**DOI:** 10.1371/journal.pone.0049838

**Published:** 2012-12-20

**Authors:** Liliana B. Menalled, Andrea E. Kudwa, Sam Miller, Jon Fitzpatrick, Judy Watson-Johnson, Nicole Keating, Melinda Ruiz, Richard Mushlin, William Alosio, Kristi McConnell, David Connor, Carol Murphy, Steve Oakeshott, Mei Kwan, Jose Beltran, Afshin Ghavami, Dani Brunner, Larry C. Park, Sylvie Ramboz, David Howland

**Affiliations:** 1 PsychoGenics Inc., Tarrytown, New York, United States of America; 2 CHDI Management/CHDI Foundation, Princeton, New Jersey, United States of America; Tokyo Medical and Dental University, Japan

## Abstract

Huntington’s disease (HD) is an autosomal dominant neurodegenerative disorder characterized by motor, cognitive and psychiatric manifestations. Since the mutation responsible for the disease was identified as an unstable expansion of CAG repeats in the gene encoding the huntingtin protein in 1993, numerous mouse models of HD have been generated to study disease pathogenesis and evaluate potential therapeutic approaches. Of these, knock-in models best mimic the human condition from a genetic perspective since they express the mutation in the appropriate genetic and protein context. Behaviorally, however, while some abnormal phenotypes have been detected in knock-in mouse models, a model with an earlier and more robust phenotype than the existing models is required. We describe here for the first time a new mouse line, the zQ175 knock-in mouse, derived from a spontaneous expansion of the CAG copy number in our CAG 140 knock-in colony [1]. Given the inverse relationship typically observed between age of HD onset and length of CAG repeat, since this new mouse line carries a significantly higher CAG repeat length it was expected to be more significantly impaired than the parent line. Using a battery of behavioral tests we evaluated both heterozygous and homozygous zQ175 mice. Homozygous mice showed motor and grip strength abnormalities with an early onset (8 and 4 weeks of age, respectively), which were followed by deficits in rotarod and climbing activity at 30 weeks of age and by cognitive deficits at around 1 year of age. Of particular interest for translational work, we also found clear behavioral deficits in heterozygous mice from around 4.5 months of age, especially in the dark phase of the diurnal cycle. Decreased body weight was observed in both heterozygotes and homozygotes, along with significantly reduced survival in the homozygotes. In addition, we detected an early and significant decrease of striatal gene markers from 12 weeks of age. These data suggest that the zQ175 knock-in line could be a suitable model for the evaluation of therapeutic approaches and early events in the pathogenesis of HD.

## Introduction

Huntington’s disease (HD) is a progressive, inherited neurodegenerative disorder characterized by involuntary movements, cognitive impairment and psychiatric manifestations. The onset of symptoms typically occurs in midlife, although it can range from early childhood to over 70 years of age [2]. While the pathological hallmark of the disease is the loss of medium spiny neurons in the striatum, neurodegeneration in the cortex and other brain areas is also observed [3]. HD is caused by an unstable expansion within the trinucleotide poly(CAG) tract located in exon 1 of the *huntingtin* gene leading to the production of a huntingtin protein with an expanded polyglutamine stretch near the N terminus [4]. Since the identification of the mutation responsible for the disease, numerous mouse models have been generated to investigate disease pathogenesis and therapeutic approaches preclinically, with all these models carrying an abnormal expansion of the CAG-repeat stretch derived from the human *huntingtin* gene.

Existing mouse models can be divided into 2 groups based on their genetics, described as transgenic or knock-in (KI) models. Transgenic mouse models have been generated by inserting into the complete murine genome either a fragment (e.g. R6/2 and N171-82Q models) or a full-length copy (BAC HD and YAC HD models) of the human *huntingtin* gene carrying an abnormal expansion of the CAG repeat tract [5], [6]. In contrast, KI mouse models carry expanded CAG repeats contained within the native murine *huntingtin* gene [7], [8] such that KI models more closely mimic the genetic context of patients with HD. KI models may contain either expanded CAG repeats within an unmodified murine gene (e.g. Hdh^(CAG(150))^) or a chimeric human/mouse exon 1 carrying the expanded CAG repeat region and the human polyproline region (e.g. CAG 71, CAG 94, CAG 140, and the zQ175 model described here).

The first KI models generated, with relatively low CAG repeat numbers, displayed either a mild or no behavioral phenotype [9], [10]. Subsequent models carrying longer expansions, however, showed behavioral abnormalities, with the Hdh^(CAG(150))^ and CAG 140 models receiving the most attention in behavioral testing. Motor deficits like poor rotarod performance, abnormal locomotion and exploratory behavior and abnormal gait have been consistently observed in homozygous mice of both lines, with the onset and severity of those abnormalities varying depending on the line examined, the specifics of the protocol used, and the strain background of the test mice [7], [8], [11]–[14]. In addition, cognitive and senory gating deficits have been reported in the Hdh^(CAG(150))^ line [15].

Given that homozygosity is very rare in humans, efforts have also been made to examine heterozygous mice. These efforts have met with limited success, with only relatively mild behavioral deficits observed with a late age of onset [11]–[13].

Given the inverse relationship between age of HD onset and CAG repeat length [16], an increase in the CAG repeat length of a KI mouse model would be expected to produce a more robust and earlier abnormal phenotype in both the heterozygotes and homozygotes. This would be beneficial for the evaluation of therapeutic approaches as well as for the examination of the early steps involved in HD pathogenesis.

We present here a detailed behavioral examination of a new mouse line carrying around 188 CAG repeats, the zQ175 KI, which was derived from the CAG 140 KI mouse model [1]. Our behavioral battery included both standard (rotarod, open field, procedural 2-choice swim test, and grip strength) and novel (PhenoCube) tests to comprehensively evaluate these novel mice. We also examined whether changes in the striatal gene markers that are affected in HD could be detected in these mice, and whether behavioral and transcriptional abnormalities would progress as the animals’ aged.

## Materials and Methods

A spontaneous expansion of the CAG repeat number was identified in a litter from our CAG 140 KI colony [1]. Founder mice from this litter were used to establish a novel colony, providing the animals used in this work. The first animals obtained carried around 175 CAG repeats, leading to the name zQ175, with the letter z used to identify that the line was derived from a line created by Scott Zeitlin. However, due to instability of the mutation, by the time the colony was stabilized the line carried around 188 CAG repeats. For tracking purposes, the CHDI nomenclature for this new KI line is CHDI-81003003.

### Subjects

Homozygous, heterozygous and wild type (WT) mice were generated by crossing heterozygous zQ175 mice on a C57B/l6J background. Animals were ear notched at around 10–15 days. Mice were weaned and implanted with RFID electronic chips (DataMars, OH) for identification at around 21 days. The average CAG repeat length in the primary test cohort was 186.2±15 (SEM = 1.4) for the heterozygous and 188.7±12 (SEM = 1.0) for the homozygous animals. Genotyping and CAG repeat count were determined by Laragen Inc. (Culver City, CA), from PCR of tail snips taken at 10–15 days of age. CAG repeat length and range in the other cohorts examined were comparable.

Three cohorts of mice were evaluated in the behavioral work described here, a primary test group (n = 9−12/sex/genotype) which underwent a full battery of testing and two secondary groups of animals (each: 7–16/sex/genotype) evaluated in the PhenoCube and swim tank (see Table 1 below for details).

**Table 1:** Test ages for behavioral work, broken down by test cohort.

	Age (weeks)																					
	4	5	8	9	12	13	16	17	20	21	22	24	25	28	29	30	32	33	36	37	38	58
**Cohort 1**																						
Open field in the light phase	√		√		√		√		√			√		√			√		√			
Open field in the dark phase	√		√		√		√		√				√	√			√		√			
Rearing/climbing in the light phase	√		√		√		√		√			√					√					
Rearing/climbing in the dark phase													√					√				
Grip Strength in the light phase	√		√		√		√		√			√					√					
Grip Strength in the dark phase													√					√				
Rotarod in the light phase		√		√		√		√		√					√					√		
Rotarod in the dark phase											√					√					√	
**Cohort 2**																						
PhenoCube							√															
Procedural 2 choice swim tank test																						√
**Cohort 3**																						
PhenoCube																				√		

Tissue was collected at three timepoints, at 12, 18 and 41 weeks of age, with an n = 5−12 per group. Tissue from the 12 and 18 week timepoints was collected from behaviorally naïve satellite groups, while the 41 week collection was taken from animals evaluated in the PhenoCube at 37 weeks (see cohort 3 in Table 1).

Animal care was in accordance with the United States Public Health Service Policy on Humane Care and Use of Laboratory Animals, and procedures were approved by the Institutional Animal and Use Committee of Psychogenics, Inc. (PHS OLAW animal welfare assurance number A4471-01), an AAALAC International accredited institution (Unit #001213).

### Husbandry

Mice from the primary test and satellite cohorts were housed in mixed genotype groups of 3–4 animals in OptiMICE® cages (Animal Care Systems, CO) with beta chip bedding (Northeastern Products Corp., NY). The PhenoCube test cohorts were instead housed in genetically homogenous groups of 6 to 10 in larger optiRAT® cages (Animal Care Systems, CO). All animals had *ad libitum* access to food (Purina 5001) and water except where noted. The cage environment was enriched with the addition of plastic bones, shredded paper and play tunnels. Temperature (68–76C), humidity (30–70%) and the light-dark cycle (6∶00–18∶00 EST) were controlled and monitored daily.

### Body Weight and Survival

In the main test cohort, body weight was measured weekly and all mice were examined daily for survival analysis. At 47 weeks of age, the animals underwent scheduled food restriction and were maintained at 85% of their free feeding weights by daily administration of limited quantities of food. At 75 weeks of age, these mice were returned to *ad libitum* feeding conditions and survival analysis and measurement continued.

The PhenoCube and tissue collection cohorts received *ad libitum* food at all times, but body weight data are not included here.

### Behavioral Testing (Primary Test Cohort)

Researchers were blind to the genotypes of the mice during testing. Mice were transported from their colony room in their home cages to the behavioral testing room, where they were allowed to acclimate for at least one hour prior to the beginning of the experiment.

#### Rotarod

Mice were tested over 3 consecutive days. Each daily session included a single training trial of 5 min at 4 RPM on the rotarod apparatus (Rotamex, OH). One hour later, the animals were tested for 3 consecutive accelerating trials of 5 min with the rotarod speed changing from 0 to 40 RPM over 300 s, with an inter-trial interval of at least 30 min. The latency to fall from the rod was recorded for each trial, with mice remaining on the rod for more than 300 sec removed and scored at 300 s.

#### Open field

Activity chambers (Med Associates Inc, St Albans, VT; 27×27×20.3 cm) were equipped with infrared (IR) beams. Mice were placed in the center of the chamber and their behavior was recorded for 30 min, with analysis performed on the following five measures: total locomotion, locomotion in the center of the open field, rearing rate in the center, total rearing frequency and velocity. Testing was carried out in both the light and dark phase of the diurnal cycle (see Table 1 below for details), in all cases at least 1 hour after the light change; testing in the dark phase was performed under red light, while testing in the light phase was performed in normal white light.

#### Grip strength

Mice were scruffed by the lower back and tail and lowered towards a mesh grip piece attached to a force gauge (Chatillion Force Gauge, San Diego Instruments, San Diego, CA) until the animal grabbed with both front paws. The animal was then lowered toward the platform and gently pulled straight back with consistent force until it released its grip. The forelimb grip force was recorded on the strain gauge.

#### Rearing-climbing

The rearing climbing chamber consisted of a metal wire mesh pencil holder placed upside down over a balance and a test mouse, such that the test animals’ weight was removed from the balance once it began to climb the mesh (modified from Hickey et al., 2005). Weight data was collected for 300 sec and latency to climb was automatically extracted.

#### Neurological assessment

Mice were examined for 1–2 minutes at 33 and 93 weeks of age. Scores for the following parameters were evaluated by simple observation of the test mice: head tremor, head twitch, head bobbing, head searching, body tremor, body twitch, tail tremor, tail twitch, straub tail, piloerection, shallow respiration, flattened body posture, swollen face, ptosis, irritability, seizure, urine staining, lacrimation, salivation, limb splay, catalepsy, abnormal gait, tip toe walking, slow careful movements, excessive grooming, circling, sniffing, chewing, state of dehydration, presence of an abnormal level of locomotor activity, and any observed seizures. In addition, tests of the startle, tail pinch and righting reflexes were carried out by direct manipulation of the test animals. To test the startle reflex, a small hand clicker was used to generate a loud popping noise and the following behaviors scored in the immediate aftermath: jumping, freezing, and rapid eye blinks. For the air righting reflex, each mouse was removed from the home cage and dropped from ∼30 cm to the arena floor, with the animals’ posture in the air scored. To measure the tail pinch reflex, forceps were used to gently squeeze the end of the tail.

Overall assessment for all these measures was scored as follows: A score of 0 was assigned for normal behavior (e.g. standard locomotor activity) or for the absence of abnormal features (e.g. the absence of piloerection); a score of 1 was given when mild abnormalities were observed and a score of 2 was given when severe abnormalities were observed. Rectal body temperature was also measured.

### Behavioral Testing (Secondary Cohorts)

#### Procedural 2-Choice swim test

Testing was carried out using a modified version of the rectangular swim tank described by Lione [17], with the tank measuring 76 cm×30.5 cm×30.5 cm, filled with opaque water to a depth of 6″ and maintained at 25±1°C. An escape platform was located 0.5 cm below the surface of the water at one end of the tank. On each test trial, mice were released from a set location relative to the platform in the middle of the tank, facing the tank wall closest to the experimenter and equidistant from either end of the tank, and were allowed to swim freely for up to 60 s. If an animal successfully reached the hidden platform within 60 s, it was allowed to remain there for 5 s and then removed from the tank. If an animal failed to find the platform within 60 s it would be manually placed onto the platform and allowed to remain there for 5 s. No spatial cues were provided within the tank environment, such that the animals were required to learn a procedural strategy to approach the platform, by turning in a particular direction.

Mice were given 4 blocks of 2 training trials each day with a 30–45 min inter-block interval. Entry point for each mouse alternated between trials within a block, along with the side of the tank in which the platform was placed, to eliminate any possible contribution of room cues to the choice of swim direction. The location of the platform was counterbalanced across groups. A correct choice was scored if the animal turned in the direction of the platform and successfully mounted the platform. An incorrect choice was scored if the animal swam in the direction opposite the platform. No choice was scored if the animal either did not make a choice, by swimming in the middle of the tank, or turned initially toward the platform but did not mount the platform.

#### Phenocube®

Animals were evaluated in the PhenoCube***®*** system in 72 h-test sessions, following a 16 h- water deprivation period in the home cage. Within the PhenoCube***®*** environment, behavior was automatically monitored at all times. The cages were maintained on a 12∶12 light/dark cycle, with white light during the day and red light during the night, maintaining a low subjective light level for the subjects during the night period. While inside the cage, water was only available from within the PhenoCube***®*** corners, while food was freely available on the cage floor at all times. Where possible, mice were left undisturbed during the course of experimental sessions. Two cohorts of mice were tested in the PhenoCube, one at 16 and one at 36 weeks of age, with all animals naïve to the PhenoCube environment at the start of testing.

The PhenoCube***®*** system was based on extensively customized Intellicage boxes (New Behavior AG) fitted with proprietary video analysis equipment. In all test sessions, the test animals initially received magazine training through a simple magazine training protocol, allowing them to freely retrieve water from any of the PhenoCube***®*** corners. Early in the light period on day 2, after a full night in the cage, the protocol was switched to a more stringent training protocol requiring the animals to visit specific locations to retrieve water and to alternate between potentially reinforced locations.

### Quantification of Transcripts

#### Tissue collection

At each specified age, striatal tissues were dissected and frozen on dry ice until they were processed for RNA extraction.

#### Total RNA extraction

Tissues were homogenized 2×1 min at 25 Hz in 750 µL of QIAzol Lysis Reagent (Cat # 79306, Qiagen, Valencia, CA) with TissueLyser (Qiagen, Valencia, CA) and 5 mm stainless steel beads (Cat # 69989, Qiagen, Valencia, CA). Once tissues were disrupted, samples were allowed to incubate at room temperate for 5 minutes. For the RNA extraction procedure, the manufacturer’s protocol for RNeasy 96 Universal Tissue Kit (Cat # 74881, Qiagen, Valencia, CA) for RNA isolation was followed. Briefly, 150 µL of Chloroform (Cat # C2432, Sigma-Aldrich, St. Louis, MO) was added and samples were shaken vigorously for 15 seconds followed by 3 minutes incubation at room temperature. The aqueous phase was separated from the organic phase by centrifugation at 6,000×g (Beckman Coulter Avanti J-30I), at 4°C for 15 minutes. The aqueous phase was then transferred to a new 96-well block and total RNA was precipitated with an equal volume of 70% ethanol. Total volume was then transferred to an RNeasy 96-well plate, followed by centrifuging at 6,000×g (Beckman Coulter Avanti J-30I), at room temperature for 4 minutes. Total RNA bound to column membranes was then treated with RNase-Free DNase set (Cat # 79254, Qiagen, Valencia, CA) for 30 minutes, followed by 3 washing steps with RW1 and RPE buffers (provided with RNeasy 96 Universal Tissue Kit). RNA was eluted with RNase-Free water (25 µL for striatum samples).

#### Total RNA quantification and reverse transcription

To ensure integrity, 2 µL of total RNA in conjunction with NorthernMax –Gly Sample Loading Dye (Cat # AM8551, Applied BioSystems, Foster City, CA) were subjected to electrophoresis in 1% agarose in NorthernMax –Gly 1× Gel Prep/Running Buffer (Cat # AM8678, Applied Biosystems, Foster City, CA). Good RNA integrity was determined by visualization of intact 16S and 23S ribosomal RNAs and the absence of any smearing. Furthermore, RNA was quantified using Quan-iT RiboGreen RNA Kit (Cat # R11490, Invitrogen, Carlsbad, CA) and analyzed with the SpectraMax Gemini XPS fluorescent plate reader (Molecular Devices, Sunnyvale, CA). One microgram of total RNA was reverse transcribed into cDNA with 3.2 µg random hexamers (Cat # 11034731001, Roche Applied Science, Indianapolis, IN), 1 mM each dNTP (Cat # 11814362001), Roche Applied Science, Indianapolis, IN), 20U Protector RNase Inhibitor (Cat # 03335402001, Roche Applied Science, Indianapolis, IN), 1X Transcriptor Reverse Transcription reaction buffer and 10U Transcriptor Reverse Transcriptase (Cat # 03531287001, Roche Applied Science, Indianapolis, IN) in 20 µL total volume. The reactions were allowed to proceed at room temperature for 10 minutes, at 55°C for 30 minutes, then were inactivated at 85°C for 5 minutes in GeneAmp PCR Systems 9700 thermal cycler (Applied Biosystems, Foster City, CA). cDNA samples were diluted 10 to 100 fold with RNase-Free water for qPCR analysis.

#### Quantitative PCR (qPCR)

Five microliters of the diluted cDNA were amplified with 12.5 µL 2× FastStart Universal Probe Master Rox (Cat # 04914058001, Roche Applied Science, Indianapolis, IN), 0.5 µL Universal Probe Library Probe (Roche Applied Science, Indianapolis, IN), 200 nM of gene specific primer- HPLC purified (Sigma-Aldrich, St. Louis, MO) in 25 µL reaction volume. The reactions were run on the ABI 7900HT Sequence Detection System (Applied Biosystems, Foster City, CA). qPCR conditions were initially set at 95°C for 10 minutes in order to activate the FastStart Taq DNA Polymerase followed by 40 cycles of 95°C for 15 seconds and 60°C for 1 minute. Primers and Universal Probe Library information are listed in Table 2.

**Table 2:** Qualitative Polymerase Chain Reaction (qPCR) information.

Mouse Gene	MouseGene ID	Gene Bank Accession Number	5′ Primer Sequence	3′ Primer Sequence	Universal Probe Library Number
ATP synthase subunitbeta	ATP5b	NM_016774.3	GGCACAATGCAGGAAAGG	TCAGCAGGCACATAGATAGCC	77
Eukaryotic initiationfactor 4A-II	EIF4A2	NM_013506.2, NM_001123037.1, NM_001123038.1	GCCAGGGACTTCACAGTTTC	TTCCCTCATGATGACATCTCTTT	93
Ubiquitin B	Ubc	NM_019639.4	GACCAGCAGAGGCTGATCTT	CCTCTGAGGCAGAAGGACTAA	11
Dopamine Receptor D2	Drd2	NM_010077.2	TGAACAGGCGGAGAATGG	CTGGTGCTTGACAGCATCTC	17
Dopamine- and cAMP-regulated neuronalphosphoprotein	DARPP32	NM_0144828.1	CCACCCAAAGTCGAAGAGAC	GCTAATGGTCTGCAGGTGCT	98
Cannabinoid receptortype 2	Cnr1	NM_007726.3	GGGCAAATTTCCTTGTAGCA	GGCTCAACGTGACTGAGAAA	79
cAMP and cAMP-inhibited cGMP3′,5′-cyclicphosphodiesterase 10a	PDE10a	NM_011866.2	GAAGGCTGACCGAGTGTTTC	GGGATGGAGAGAAAGATAGGC	45
Glutamatetransporter 1	GLT1	NM_001077515.2 NM_001077514.3 NM_011393.2	GGTCATCTTGGATGGAGGTC	ATACTGGCTGCACCAATGC	83

#### qPCR data analysis

Total RNA from whole brain of C57BL6 mice was reverse transcribed as described above. cDNA samples from multiple reverse transcription reactions were pooled together and used to create qPCR standard curves for the genes of interest and also served as the calibrator, diluted just as the sample cDNA, to normalize plate to plate variations. To generate the standard curve, pooled cDNA was serial diluted from 1∶5 to 1∶1000 in RNase-free water and assayed in triplicate in each qPCR assay. The Ct values (number of cycles required for the PCR amplicon detection to reach threshold) were plotted against the logarithm value of dilution samples and a linear trend line was obtained for each gene. PCR efficiencies were calculated using the equation of PCR efficiency = 10^(1/−slope)^. Note that a PCR efficiency of 2 is the value for ideal PCR amplification where the amplicons double in quantity per PCR cycle.

Each sample of cDNA (diluted 1∶10) was assayed in triplicate and the Ct values averaged. Values which lay greater than 0.5 standard deviations from the average were discarded. The relative quantity of the PCR product (relative to the calibrator) is calculated as follows:

Relative Quantity of Target gene = (PCR Efficiency_Target_)^(Ct calibrator-Ctsample)^.

Relative Quantity of Housekeeping Gene 1 =  (PCR Efficiency_housekeeping1_)^(Ct calibrator-Ctsample)^.

Relative Quantity of Housekeeping Gene 2 =  (PCR Efficiency_housekeeping2_)^(Ct calibrator-Ctsample)^.

Relative Quantity of Housekeeping Gene 3 =  (PCR Efficiency_housekeeping3_)^(Ct calibrator-Ctsample)^.

The geometric mean for the three housekeeping genes is calculated as follows:

Geometric mean = (relative quantity of housekeeping gene 1 * relative quantity of housekeeping gene 2 * relative quantity of housekeeping gene 3)^(1/3)^.

The relative level of the target gene is calculated as follows:

Relative Quantity of Target gene/Geometric mean of housekeeping genes.

The 3 striatal housekeeping genes used in this study were: *ATP5b*, *Eif4a2* and *Ubc*. The relative level of the target gene (*Drd2*, *DARPP32*, *Cnr1*, *PDE10a* and *GLT1*) was then normalized to age and gender matched wild type control animals.

### Data Analysis

An alpha level of.05 was selected for all inferential statistics. Repeated measures analysis (age as dependent factor) was carried out with SAS (SAS Institute Inc.) using Mixed Effect Models. This approach was based on likelihood estimation which was more robust to missing values than moment estimation. The models were fitted using the procedure PROC MIXED [18]. For parametric analyses, genotype, gender, age and their interactions were considered in all the models and significant genotype × age, genotype × gender and genotype × age × gender interactions were followed up with simple main effects to determine at which age the genotype differences reached significance.

For PhenoCube***®*** testing, data were summarized across the two full day/night periods obtained after the mice had fully acclimated to the PhenoCube***®*** environment. Data collapsed from these two test periods was then evaluated via ANOVA as outlined above, though line graphs are also presented describing the full experimental sessions in shorter time bins, to provide a visual representation of the animals’ behavioral patterns. For these PhenoCube analyses, an additional factor of day/night cycle was included, capturing activity differences across the animals’ activity cycles.

Rearing and climbing data were analyzed using Chi square analysis (StatView), seperately comparing the WT group to homozygous and to heterozygous animals. The proportion of mice to reach the acquisition criterion in the swim test task was assessed using Kaplan-Meier event analysis over the entire acquisition test period, followed up with Chi square analysis of the effects of genotype on performance on individual test days.

The neurological assessment data were analyzed using Chi square analysis.

Survival data were analyzed with Kaplan–Meier analysis, with the *p* values derived from the Mantel–Cox Log-rank statistic.

## Results

### Behavioral Evaluation

#### General health and neurological abnormalities

At 33 weeks of age, body tremor was observed in 68% of the homozygous mice but in only 4% in the WT group (χ^2^ test, *p*<0.05). 21% of homozygous mice were found to be hypoactive, while locomotor activity was normal in all WT mice at this age (χ^2^ test, *p*<0.05). In contrast to homozygous mice, tremors were observed in only 13% of the heterozygous mice, not differing significantly from the WT controls, with no apparent hypoactivity observed at this age (χ^2^ tests, *ps* >0.05). When body temperature was evaluated, homozygous mice presented a significantly lower body temperature than WT animals (*p*<0.05), with no difference detected between heterozygous and WT animals (Mean values ± SEM: WT, 36.12°C ±0.54; heterozygous, 35.76°C ±0.64; homozygous: 35.60°C ±0.75; Genotype effect F(2,63) = 3.784, *p*<0.05).

At 93 weeks of age, neurological assessment revealed that a high percentage (between 80 and 100%) of the surviving homozygous mice presented with loss of air righting reflex, body tremor, abnormal gait, limb splay, hunched posture, flattened body posture and piloerection, differing significantly from WT controls (χ^2^ test, *ps* <0.05). Interestingly, features of pre-seizure activity, such as body twitches, straub tail and partial praying seizure activity without rearing, were observed in most of the homozygous mice (>80% of animals). An abnormal startle response and poor grooming were observed in 66% and 50% of the homozygous mice examined (χ^2^ test, *ps* <0.05). Heterozygous mice at 93 weeks also presented significantly abnormal body tremor (70% of mice), startle response (∼80%), piloerection (85%) and abnormal gait (including limb splay and hunched posture; 90–100% of mice) relative to WT control animals (χ^2^ test, *ps* <0.05). Overall neurological abnormalities appeared more severe in the homozygous than in the heterozygous animals. As at 33 weeks, the homozygous mice again presented a significantly lower body temperature at 93 weeks of age than either WT or heterozygous mice animals (*ps* <0.05), with no difference in body temperature detected between heterozygous and WT animals (WT: 36.78°C ±0.56; heterozygous: 36.49°C ±0.92; homozygous: 35.45°C ±0.72; F(2,44) = 7.04, *p*<0.05). No seizures were observed at any age either spontaneously or during handling/behavioral testing.

#### Loss of body weight in both heterozygous and homozygous zQ175 KI mice

Homozygous female and male mice gained body weight at the same rate as WT mice up to 7 and 5 weeks of age, respectively. Subsequently, the homozygous animals were significantly lighter then WT controls. While the body weight of female homozygous mice remained constant during the evaluation period, male homozygous body weight first plateaued and then progressively decreased with increasing age. Homozygous mice were also significantly lighter than heterozygous mice from 4 weeks onward in the male group and from 19 to 32, from 34 to 36 and at 38 weeks of age in the female group.

**Figure 1:**
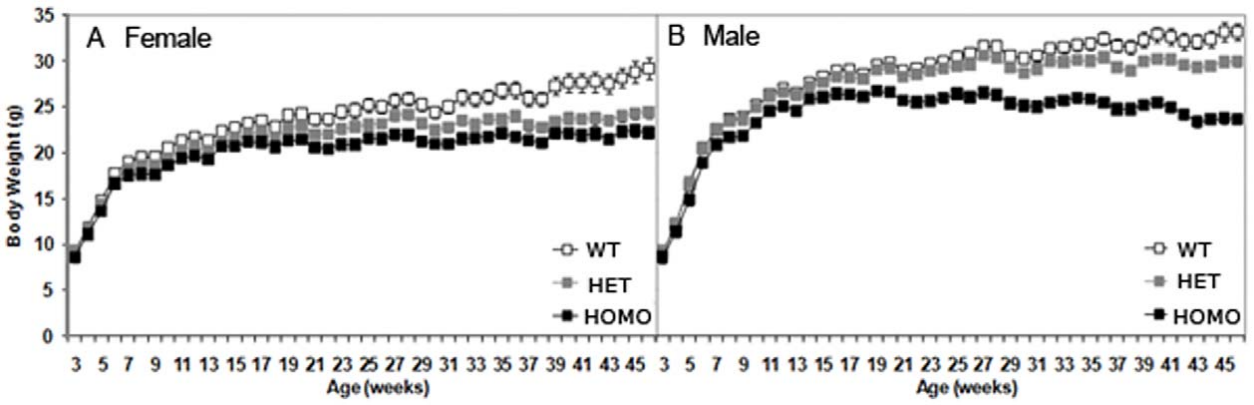
Body weight (mean ± SEM) of wild type, heterozygous and homozygous mice as a function of age for female (A) and male (B) mice.

Heterozygous zQ175 mice were also significantly lighter than WT controls, but this phenotype was observed at later ages than in the homozygous mice, being apparent only at 26 and from 28 weeks of age onwards in the male group and at 10, 13 and from 15 weeks of age onwards in the female group (Figure 1, genotype main effect, F(_2,61_) = 41.60, *p*<0.0001; age main effect, F_(8,2294)_ = 212.59, *p*<0.0001; genotype × age interaction, F_(76,2294)_ = 2.37, *p*<0.0001; genotype × age × gender interaction, F_(114,2294)_ = 3.04, *p*<0.0001; simple main effects *ps*<0.05).

#### Reduced survival in zQ175 homozygous mice

**Figure 2:**
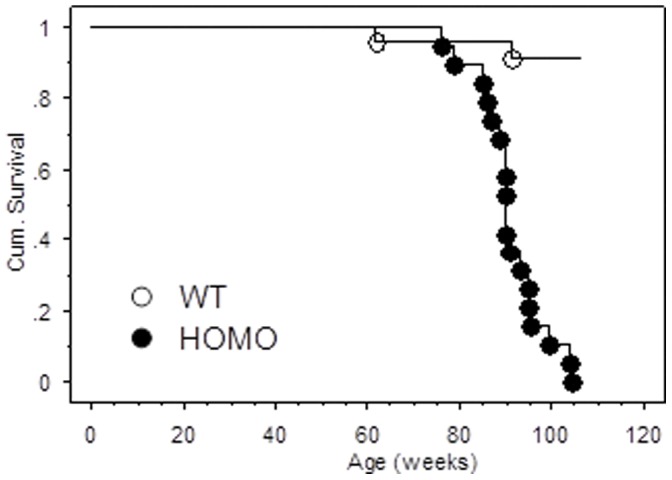
Kaplan-Meier survival curve in WT vs. homozygous mice as a function of genotype and age.

Survival differences began to appear relative to WT controls in the homozygous group at around 76 weeks of age (see Figure 2), with all homozygous mice dead by 104 weeks of age with a median survival of 90.1 weeks (WT vs. homozygous Kaplan-Meier Mantel-Cox: *p*<0.0001). At this age (104 weeks), only around 25% of the heterozygous mice and 8% of the WT mice had died, with no analysis performed on the WT vs. heterozygous comparison.

#### Hypoactivity in the dark phase is seen in open field testing: Total distance

**Figure 3:**
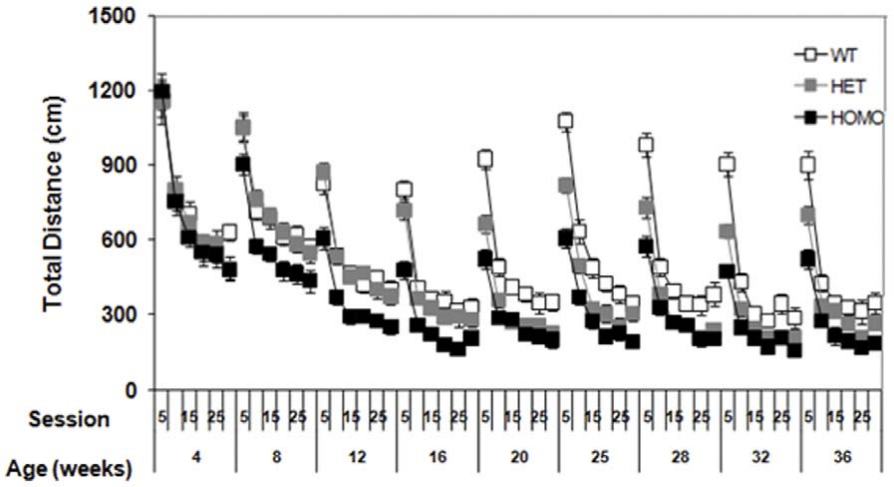
Total distance covered in the Open Field per 5 minute bin (mean ± SEM) of wild type, heterozygous and homozygous mice as a function of age and test time during the dark phase of the diurnal cycle.

Mice were evaluated during the light and dark phase of the diurnal cycle in open field to assess motor function, general activity, reaction to a novel environment, and exploration mice. In the testing during the dark phase of the diurnal cycle, homozygous mice presented a decreased total distance travelled relative to WT controls from 8 weeks of age. While the same phenotype was observed in heterozygous animals, onset was much later, with hypoactivity seen only from 20 weeks of age onwards. In addition, the homozygous mice were significantly more hypoactive than were heterozygous mice at 8, 12, 16, 25 and 36 weeks of age (genotype main effect, F_(2,61)_ = 29.01, *p*<0.0001; age main effect, F_(8,483)_ = 142.32, *p*<0.0001; genotype × age interaction, F_(16,483)_ = 3.74, *p*<0.0001; simple main effects *ps*<0.05, Figure 3).

#### Hypoactivity in the dark phase is seen in open field testing: Rearing

**Figure 4:**
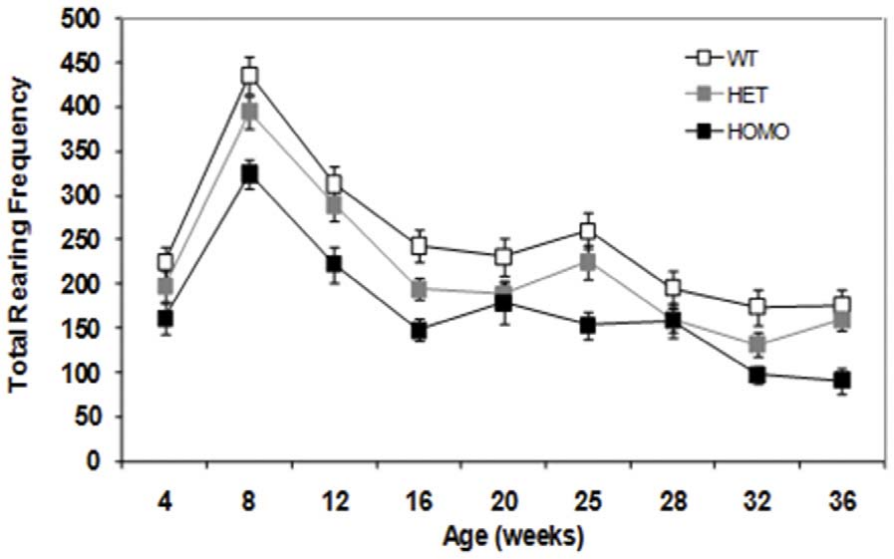
Rearing frequency in the Open Field (mean ± SEM) of wild type, heterozygous and homozygous mice as a function of age during the dark phase of the diurnal cycle.

Homozygous mice reared significantly less than WT mice during the dark phase of the diurnal cycle at all ages except at 4 and 28 weeks of age. While there was an evident decrease in locomotor and rearing activity of the homozygous mice as they aged, this was paralleled by decreases in the activity of the WT control group, producing an overall genotype effect and precluding evaluation of the progression of motor deficits using those parameters. Heterozygous mice reared significantly less than WT controls at 16 and 32 weeks of age and more than homozygous mice at 12, 16, 25 and 32 weeks of age (genotype main effect, F_(2,61)_ = 11.02, *p*<0.0001; age main effect, F_(8,483)_ = 52.82, *p*<0.0001; genotype × age interaction, F_(16,483)_ = 2.60, *p*<0.0008; simple main effects *ps*<0.05, Figure 4).

#### Hypoactivity is also seen in the light phase in open field testing: Total distance

Similarly, testing in the light phase of the diurnal cycle revealed robust hypoactivity in the female homozygous mice relative to WT controls starting at 8 weeks of age. However, in the light period, no significant differences were observed between the heterozygous female and WT female animals, though homozygous female mice were hypoactive relative to the heterozygous female mice at 8, 12, 20, 28 and 32 weeks of age. In the male group, homozygous and heterozygous mice both presented significantly decreased locomotor activity relative to WT males only at 20 weeks of age (Figures S1A and S1B, genotype main effect, F_(2,61)_ = 9.75, *p*<0.0003; age main effect, F_(8,482)_ = 86.79, *p*<0.0001; genotype × age interaction, F_(16,482)_ = 2.02, *p*<0.02; genotype × gender × age interaction, F_(24,482)_ = 2.82, *p*<0.0001; simple main effects *ps*<0.05).

#### Hypoactivity is also seen in the light phase in open field testing: Rearing

Homozygous mice reared significantly less than did WT controls mice during the light phase of the diurnal cycle at all ages, regardless of gender (Figure S2, genotype main effect, F_(2,61)_ = 3.58, *p*<0.04; age main effect, F_(8,482)_ = 42.0, *p*<0.0001). No significant differences were detected when comparing the heterozygous animals to either homozygous or WT mice.

#### Motor deficits are seen in both homozygous and heterozygous mice: Rotarod performance

**Figure 5:**
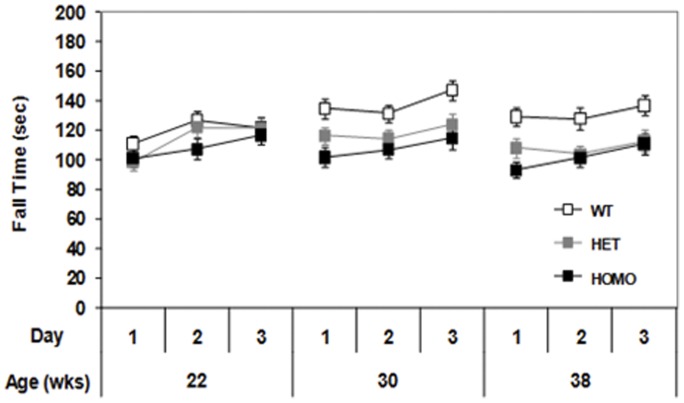
Latency to fall from the rotarod (mean ± SEM) of wild type, heterozygous and homozygous mice as a function of age during the dark phase of the diurnal cycle.

To assess motor coordination, mice were tested using the accelerating rotator protocol. During the diurnal dark period, both homozygous and heterozygous mice showed a reduced latency to fall from the rotarod relative to WT control mice at 30 and 38 weeks of age (Figure 5, genotype main effect, F_(2,60)_ = 5.13, *p*<0.01; age main effect, F_(2,120)_ = 9.69, *p*<0.0001; genotype × age interaction, F_(4,120)_ = 6.28, *p*<0.0001; simple main effects *ps*<0.05). No significant deficits were detected in the light phase (data not shown).

#### Deficits in grip strength performance were detected in homozygous mice

**Figure 6:**
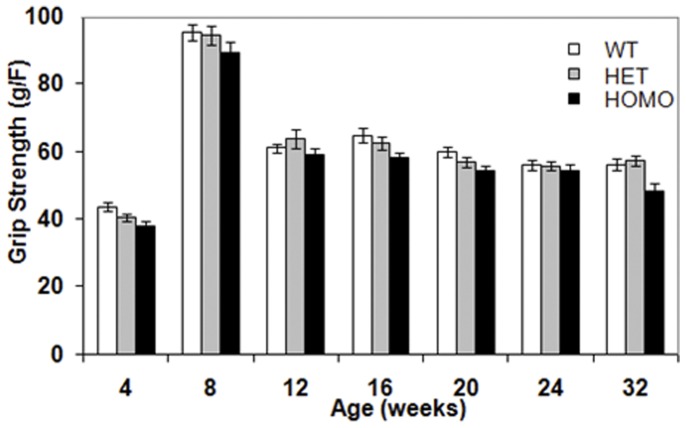
Grip strength (mean ± SEM) of wild type, heterozygous and homozygous mice as a function of age during the light phase of the diurnal cycle.

To assess muscle strength, mice were tested in the grip strength test.At all ages and genotypes, female mice showed weaker grip strength than did males. The homozygous mice presented a decreased grip strength performance than did WT controls at all the ages evaluated in both the light and dark phases of the diurnal cycle, with testing starting at 4 weeks of age (light phase data not shown: genotype main effect, F_(2,61)_ = 9.39, *p*<0.0005; gender main effect, F_(1,61)_ = 28.12, *p*<0.05, age main effect, F_(6,363)_ = 218.42, *p*<0.0001; simple main effects, *ps* <0.05, Figure 6: dark period data: genotype main effect, F_(2,60)_ = 6.56, *p*<0.005; gender main effect, F_(1,60)_ = 5.49, *p*<0.04, age main effect, simple main effects, *ps* <0.05, Figure S3).

#### Decreased climbing activity was observed in homozygous mice

**Figure 7:**
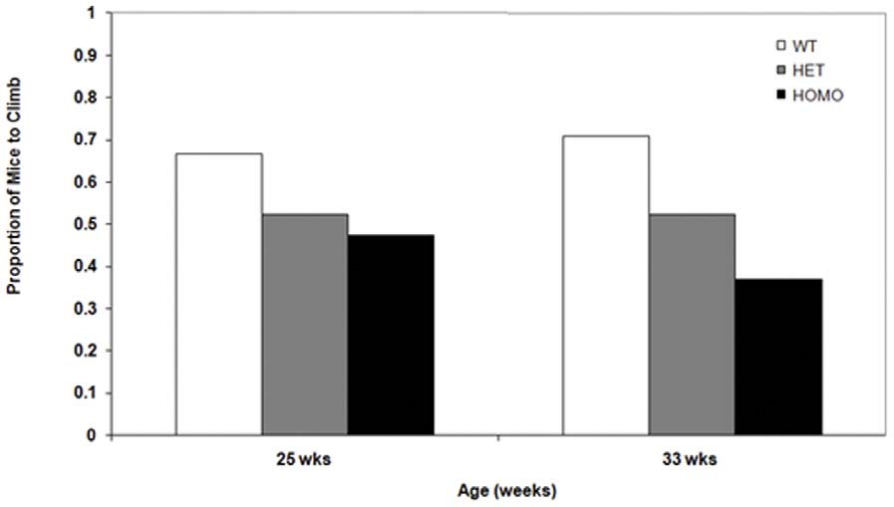
Proportion of mice climbing in the rearing climbing assay in the wild type, heterozygous and homozygous group during the dark phase of the diurnal cycle.

To assess climbing activity mice were evaluated in the rearing-climbing assay. A smaller proportion of mice in the homozygous group climbed during the dark phase of the diurnal cycle at 33 weeks of age (Figure 7, χ^2^ test, *p*<0.05) but not at 25 weeks of age. Decreased climbing activity relative to WT mice was observed in the homozygous animals during testing in the light phase at 32 weeks of age, but not at younger ages(data not shown). No deficits were detected in the heterozygous mice.

#### Cognitive deficits in homozygous mice

**Figure 8:**
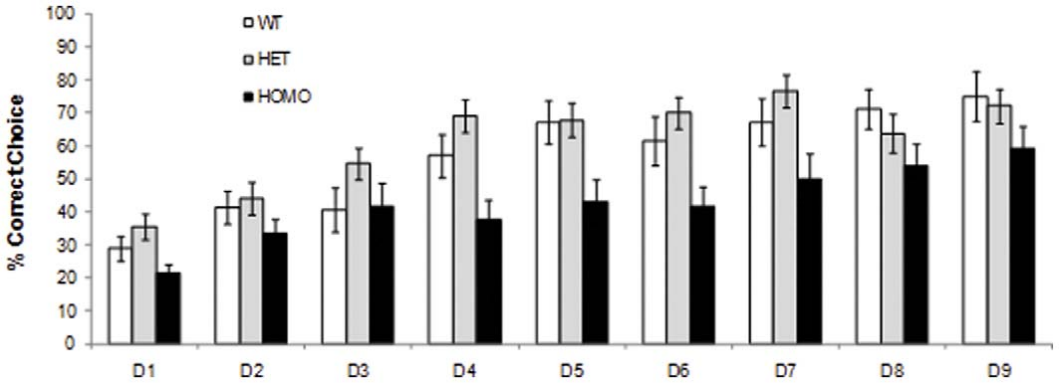
Percent correct choices for WT, heterozygous and homozygous mice per test day during acquisition of the procedural swim tank at 58 weeks of age.

To assess learning and memory, mice were tested in the procedural two-choice swim test, a simple left-right discrimination task. Homozygous mice performed significantly less well than either heterozygous or WT mice in overall acquisition of the procedural swim tank task at 58 weeks, with a significantly lower percentage of correct choices recorded during acquisition (genotype main effect: F_(2,58)_ = 9.15, *p*<0.001; simple main effects *ps*<0.05; Figure 8) as well as a significantly higher level of incorrect choices (genotype main effect: F_(2,58)_ = 8.89, *p*<0.001; simple main effects *ps* <0.05, data not shown). Choice latencies were also significantly higher in homozygous mice than in either heterozygous or WT mice (genotype main effect: F_(2,58)_ = 58.60, *p*<0.0001; genotype × day interaction: F_(16,464)_ = 3.28, *p*<0.0001; simple main effects *ps*<0.05, data not shown).

#### PhenoCube®

PhenoCube is a high-throughput platform that assesses circadian, cognitive and motor behavior exhibited by group-housed mice. Data are presented here from two separate cohorts of naïve animals. The initial cohort was tested at 16 weeks of age, while the second cohort was tested first at 36 weeks of age. Prior to testing, animals were minimally disturbed, living in their testing groups in the colony rooms. In some cases, animals failed to record licks within the PhenoCube® corners and consequently were removed from testing – no data from these mice are presented.

#### PhenoCube® – visit frequency

**Figure 9:**
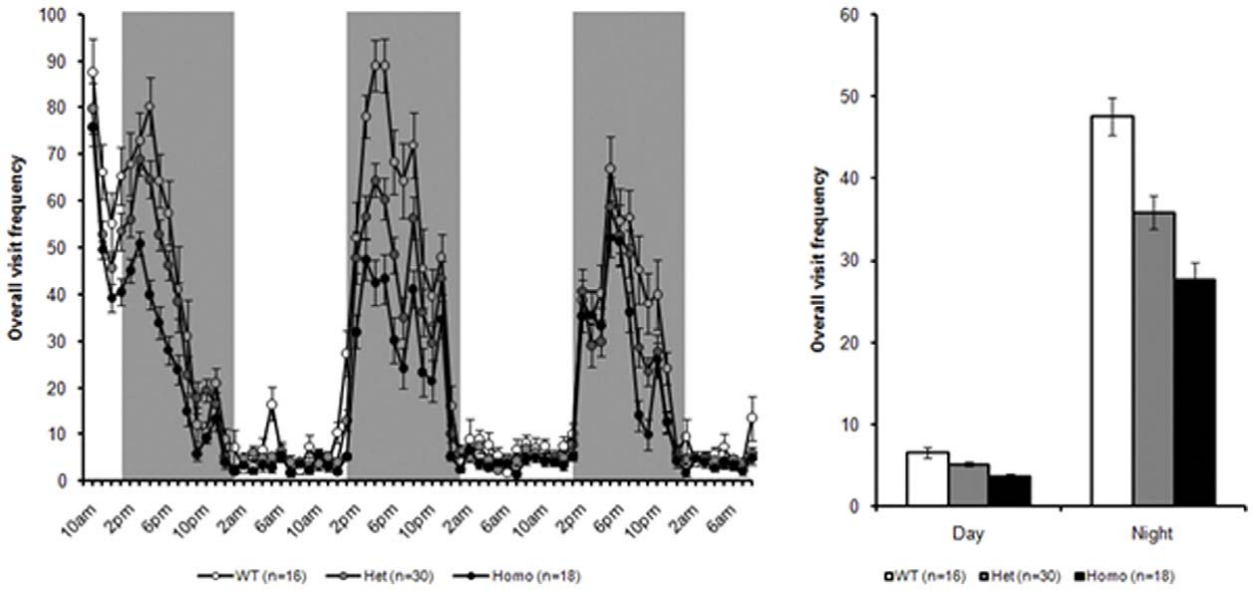
Overall visit frequency during PhenoCube testing at 16 weeks, broken down into 1 hour bins (A) and summarized across the two complete light/dark periods from lights-on on day 2 (B).

**Figure 10:**
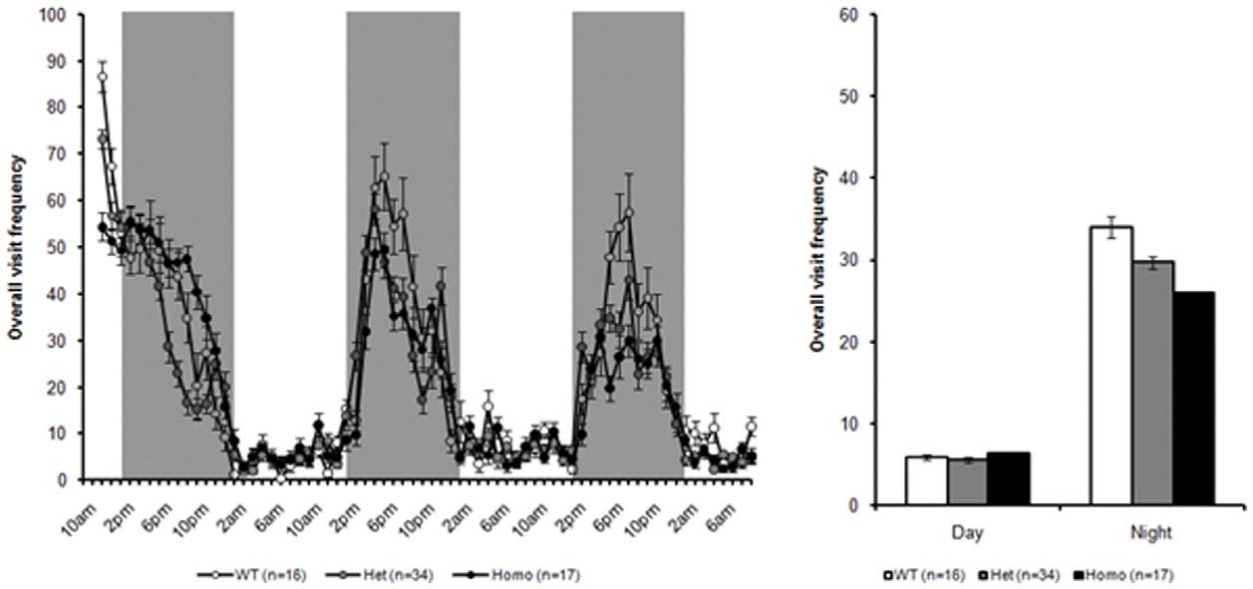
Overall visit frequency during PhenoCube testing at 37 weeks, broken down into 1 hour bins (A) and summarized across the two complete light/dark periods from lights-on on day 2 (B).

Inspection of these data indicated that all mice show markedly increased activity in the night vs. the day periods, with genotype differences consequently became more apparent during the night time. There appeared to be clear differences between all three genotypes at both ages, though the effects were clearer in the 16 week test age (Figure 9, than at 37 week of age (Figure 10) as the WT animals become less active with age. Consistently, there were significant main effects of both test age and day/night cycle, smaller F_(1, 119)_ = 12.9, along with a significant overall effect of genotype, F_(2, 119)_ = 23.3, all *ps <0.0001*. There were also significant interactions amongst these factors, with a significant genotype × test age and a significant genotype × day/night cycle interaction, smaller F_(2, 119)_ = 5.44, along with a significant three way interaction between genotype, test age and day/night cycle, F_(3, 119)_ = 9.18, all *ps <0.01*. Follow-up analysis confirmed that all three genotypes were statistically distinct in the night period at both ages, with the zQ175 heterozygous mice less active than WT controls but more active than zQ175 KI homozygous mice, smallest t_(119)_ = 2.00, *ps <0.05*. More detailed breakdown of these statistics is presented in Appendix S1, below.

#### Level of expression of striatal transcripts

To evaluate whether striatal mRNA expression was affected by zQ175 genotype, the mRNA expression levels of dopamine D2 receptor (*Drd2*), phosphodiesterase-10a (*PDE10a*), cannabinoid receptor-1 (*Cnr1*), dopamine-and cyclic-AMP-regulated phosphoprotein (*DARPP32*), and glutamine transporter-1 (GLT1) were assessed. Striatal samples from homozygous and heterozygous mice were examined at 12 (males and females), 18 (male only), and 41 (males and females) weeks of age. The geometric mean of three housekeeping genes, *ATP5B*, *EIF4A2*, and *Ubc*, as has been previously described [19] was used to normalize the transcripts of interest. In order to better evaluate the validity of the disease marker changes, we decided to first examine the degree of housekeeping gene variability by normalizing each housekeeping gene to the geometric mean of itself and two other housekeeping genes (data not shown). This exercise allowed us to better interpret whether changes obtained with the genes of interest are to the same extent or superior to those of normalized housekeeping genes. It is important to note that in most cases, housekeeping genes did not show either genotypic or age related changes (data not shown).

#### Transcript abnormalities were seen in both heterozygous and homozygous mice

**Figure 11:**
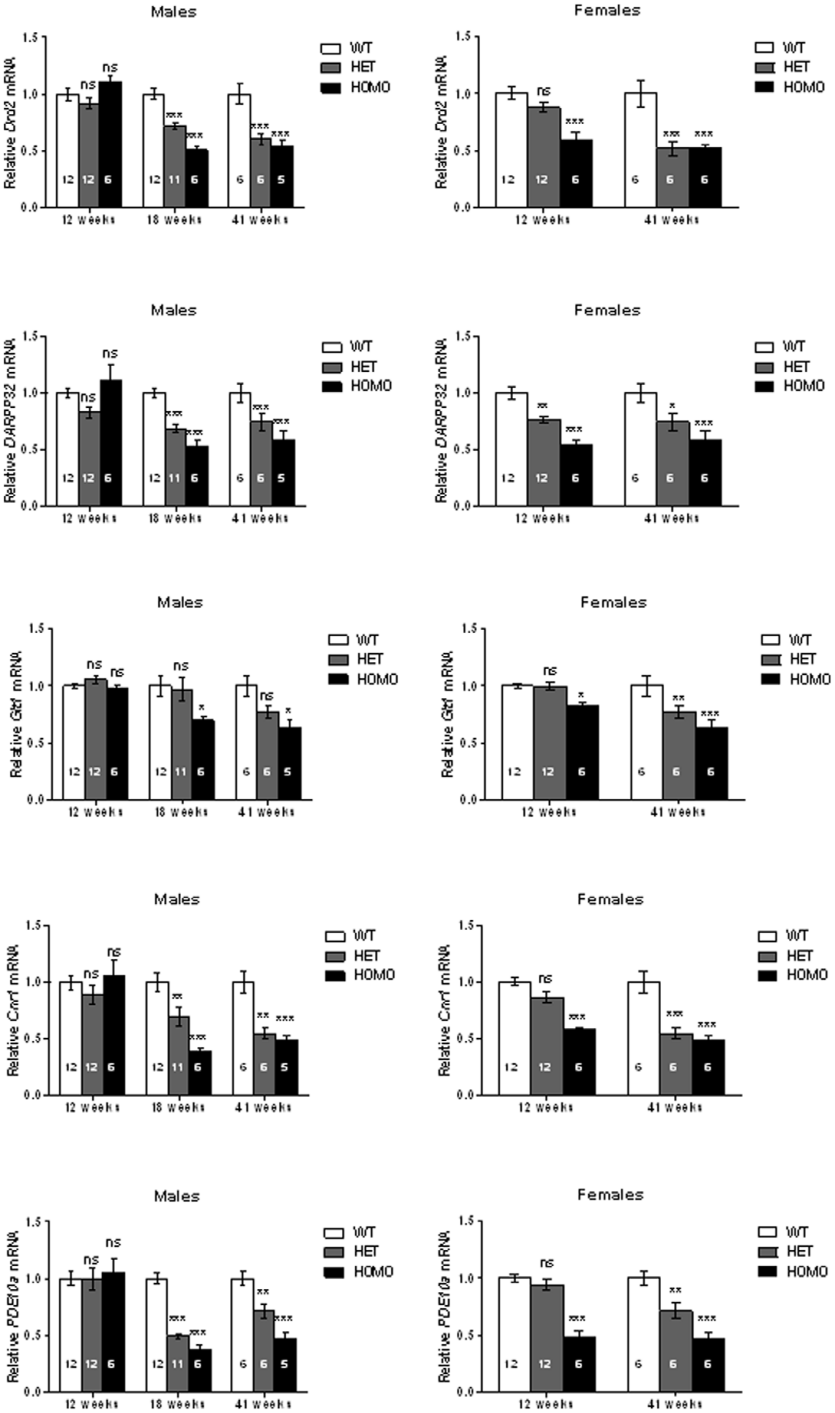
The relative striatal mRNA expression level of wildtype (WT), heterozyous (HET) and homozygous (HOMO) zQ175 mice at 12, 18 and 41 weeks of age, analyzed by qPCR. Relative mRNA levels are normalized to age matched and gender matched wild type controls. For normalization, the geometric mean of UBbc, Eif4a2 and ATP5B was used. Gender separation was performed with the 12 and 41 week groups. *, p<0.05; **, p<0.01; ***, p<0.001. The number on each bar graph represents “n” for each group.

We detected transcript level differences between the genotypes and sexes. In 12 weeks old female heterozygous mice, only *DARPP32* mRNA level was significantly reduced (post hocs, *ps* <0.05*;* Figure 11; Table S1 presents the F and *p* values for the significant main effects and interactions for the different gene markers examined for male and female mice), whereas in 12 week-old female homozygous mice a significant reduction in expression level of all transcripts reported in this study was observed (*ps* <0.05). In males, however, transcript downregulation in both homozygous and heterozygous mice occurred around 18 weeks of age with a significant downregulation of *Drd2*, *DARPP32*, *Cnr1* and *PDE10A* mRNA levels (*ps* <0.05). At this age, GLT1was the only transcript that showed genotype differences since a significant reduction of expression was detected in 18 weeks old male homozygous but not in heterozygous mice (*ps* <0.05). An age dependent decrease of mRNA expression level has been also been noted. At 41 weeks of age, *Drd2*, *DARPP32*, *Cnr1* and *PDE10a* transcriptional levels were significantly reduced in both males and females of heterozygous and homozygous mice, with the greatest reduction (about 50%) seen in homozygous mice relative to their WT littermates (*ps* <0.05). In addition at 41 weeks of age, *GLT1* mRNA expression level was not only reduced in female homozygous, but at this age a significant downregulation was also noted in both homozygous male and heterozygous female mice.

## Discussion

Novel HD mouse models that present a more robust and earlier onset of phenotype and that more closely mimic human HD are required. We present here a behavioral and molecular characterization of a new KI HD mouse model, the zQ175 KI line, that carries around 188 CAG repeats. This line arose spontaneously in a litter carrying around 175 CAG repeats in our CAG 140 mouse colony (Menalled, Sison et al. 2003); due to the instability of the mutation and multiple rounds of breeding, we developed a colony carrying around 188 CAG repeats. Careful selection from a large pool of potential breeder animals thereafter allowed us to maintain the line with around 188 CAG repeats, as in the cohorts used here. We show that homozygous mice present robust motor and cognitive abnormalities, many of them with an earlier onset than reported in other KI models. Data from the PhenoCube® platform confirm that motor abnormalities detected in both homozygous and heterozygous mice were not related to handling stress, since clear hypoactivity was observed when the animals were spontaneously active. Decreased body weight was detected in both heterozygotes and homozygotes, along with reduced lifespan in the homozygotes. Abnormalities were also observed in striatal mRNA expression. Critically, both the behavioral and transcriptional phenotype observed in these mice appeared at younger ages than in previously described models, particularly in the heterozygotes. Importantly, power analysis shows that group sizes of 4–15 mice for most of the transcripts analyzed are sufficient to detect a 50% improvement (final number needed depends on age, genotype and sex of interest; α = 0.05, 80% power, see Table S2 for details). A sample size of 15 animals also allows the evaluation of potential therapies in HOM mice using a general heath and motor behavioral battery and of 35 to include the cognitive domain (for more details see Table S3). Deficits in the HET mice are not as pronounced compared to the deficits detected in the HOM group. Sample sizes of 5–22 HET mice are sufficient to detect a 50% improvement in most of the transcripts analyzed (Table S2). A sample size of 37 HET mice allows us to detect an effect size of at least 50% in body weight and in the total distance in the open field test (first 5 min) when performed during the dark phase of the diurnal cycle (Table S3). Together, our data indicate that these zQ175 KI mice represent a valuable new preclinical model of HD.

### General Health Abnormalities

Loss of body weight is a progressive and characteristic symptom of HD [20]. Decreased body weight has been observed in numerous mutant mouse and rat models of HD [21], [22] and was recapitulated in the new model described here. Significantly, the body weight of the heterozygous zQ175 mice was intermediate between that of the homozygous and WT mice, in a manner consistent with previous findings that lower levels of WT full-length huntingtin are associated with decreased body weight (Van Raamsdonk et al., 2006).

Given the inverse relationship typically seen between the age of onset of HD phenotype and the CAG repeat length, it is perhaps surprising that these zQ175 mice present a significantly earlier onset of body weight abnormalities than were recently reported in a KI mouse line carrying 200 CAG repeats, the HdhQ200 model [8]. Since the CAG repeat length of the zQ175 and HdhQ200 lines are comparable, our results highlight that differences in age of onset of body weight phenotype in KI HD mouse models are not exclusively influenced by CAG repeat length, but are also affected by background strain, colony conditions, and genetic construct used [23]. A progressive decline in the animals’ general health was detected in both the homozygous and heterozygous zQ175 KI mice. Specifically tremors, hypoactivity, abnormal gait, and flattened body posture were more prevalent as the animals aged. While these abnormalities are consistent with those seen in other KI mice expressing similarly long CAG repeats, none of those models presented these type of deficits at such an early age (from 33 weeks) [8], [11], [14], [24]. Furthermore, these zQ175 homozygous mice are the first KI animals in which a significantly reduced lifespan has been reported [1], [8], [14]. Given that the mice described here were food restricted from 47 to 75 weeks of age, some caveats might exist with these survival data, but it is worth noting that the survival curve observed here up to 58 weeks is very similar to that of a second cohort evaluated in our laboratory that did not undergo food restriction (unpublished observation).

Reduced grip strength is seen in HD patients and also in a number of HD mouse models [25], [26]. Decreased forelimb grip strength was detected from 4 weeks of age in these homozygous zQ175 mice and has previously been reported at around the same age in the Hdh^(CAG(150))^ homozygous KI mouse (Woodman, Butler et al. 2007).

### Motor Abnormalities

Motor abnormalities, cardinal features of the human condition, are also apparent in many HD mouse models [1], [8], [12], [14], [15], [27]. We used four behavioral methods to evaluate motor decline; rotarod performance, open field activity, climbing behavior, and the PhenoCube® system. Testing in the diurnal dark phase when spontaneous activity was higher (illustrated by the PhenoCube data) showed genotype-dependent deficits that otherwise might not have been observed. Whilst the basic locomotor and rearing abnormalities detected in this new line have an early onset and are robust, especially in the homozygotes, the deficits in those parameters did not seem to progress with age (at least as examined, up to 36 weeks). It is possible that these mice might progress at older ages, though age-related decline in performance of the WT mice may complicate analysis.

Similarly, rotarod deficits were observed both in the heterozygous and homozygous zQ175 mice from 30 weeks of age, but no progression of these deficits was observed with age, again as with previous observations in CAG 140 mice [13], [14]. Our results warrant further examination in, for example, a beam test and running wheels that have shown progression in other KI mouse models [8].

In PhenoCube® testing, significant hypoactivity was detected in both heterozygotes and homozygotes at the ages tested (16 and 36 weeks), with significantly reduced activity also observed in homozygotes relative to heterozygotes.

A moderately-enriched environment is part of the regular housing conditions for HD mouse models in our facility, and such environments can ameliorate HD phenotypes in mouse models as well as patients [28]–[30]. The early phenotype we detected in this new KI line (relative to other KI lines) occurred despite this enriched environment, again highlighting the robustness of the phenotype in the zQ175 line.

### Cognitive Abnormalities

Cognitive decline is a key feature of HD, with a very specific pattern of progressive deficits predominantly related to damage to the frontal-striatal circuitry, and this damage is recapitulated in many HD mouse models. We trained zQ175 mice in a simple procedural response learning that should be impaired by striatal dysfunction and which had previously revealed very substantial deficits in the R6/2 mouse (unpublished observations). We found that zQ175 homozygotes presented deficits in this task at 58 weeks, while there were no differences between the heterozygous and WT mice. Previous accounts of cognitive deficits in Hdh^(CAG(150))^ homozygous KI mice indicated impairments in demanding tasks (i.e. reversal learning and extradimensional shifts) apparent as early as 4–6 months of age while no deficits were detected in simple response learning tasks [15], [31].Work in our laboratory has revealed significant deficits in zQ175 heterozygous mice in a more complex visual discrimination reversal task with a touchscreen [32], [33]. Furthermore, work in the R6/2 transgenic HD mouse has similarly demonstrated that more complex learning deficits (e.g. reversal) can be identified at earlier ages than simpler learning deficits (e.g. acquisition) [17]. The significant deficit in simple procedural response learning in zQ175 mice at 58 weeks of age may therefore be the result of either their advanced age or their higher CAG repeat expansion, and warrants further evaluation of younger zQ175 cohorts in this and other cognitive assays to better define the nature and breadth of impairment.

### Transcriptional Dysfunction

Previous studies in both humans and mouse models of HD indicated that transcript levels of specific genes in the striatum are reduced [34]–[44]. We also show here that expression levels of previously reported striatal transcripts such as dopamine D2 receptor (*Drd2*), phosphodiesterase-10a (*PDE10a*), cannabinoid receptor-1 (*Cnr1*), dopamine-and cyclic-AMP-regulated phosphoprotein (*DARPP32*), and glutamine transporter-1 (*GLT1*), were also reduced in zQ175 mice. Further study will determine whether this downregulation is due to neuronal loss or dysfunction; it is unlikely that loss is the only cause since the expression of housekeeping genes was unaltered.

In general, behavioral deficits in zQ175 mice emerge at about the same age as do transcriptional abnormalities, with homozygotes showing a phenotype earlier than heterozygotes. It is important to note that the differential onset of the phenotype in heterozygotes vs. homozygotes in this report and a previous study [13] do not translate to observations in HD patients; patients either homozygous or heterozygous for the mutant huntingtin gene typically have a similar onset of disease symptoms and differentiate only later in disease progression [45]. Interestingly, the onset of transcript downregulation in male mice is the same for both the homozygotes and heterozygotes (18 weeks).

### Summary

In summary, we describe here a new zQ175 KI mouse model that closely mimics the human genetic lesion and presents robust and early behavioral and molecular alterations in both homozygous and heterozygous mice. Further characterization will determine its utility as a suitable model for the study of HD pathogenesis, as well as for the evaluation of therapeutic approaches.

## Supporting Information

Figure S1 **Total distance travelled (mean ± SEM) of wild type, heterozygous and homozygous zQ175 mice as a function of age for female (A) and male (B) mice during the light phase of the diurnal cycle.**
(DOCX)

Figure S2 **Rearing frequency (mean ± SEM) of wild type, heterozygous and homozygous mice as a function of age during the light phase of the diurnal cycle.**
(DOCX)

Figure S3 **Average grip strength (mean ± SEM) of wild type, heterozygous and homozygous mice as a function of age during the dark phase of the diurnal cycle.**
(DOCX)

Table S1 **F and p value table for the significant main effects and interactions for the different gene markers examined for male and female mice.**
(DOCX)

Table S2 **Summarizes the sample size needed to detect a 50% effect in the transcripts evaluated with an alpha of 0.05 and a power of 0.8 for the HET and HOM mice.**
(DOCX)

Table S3 **Summarizes the sample size needed to detect a 50% effect in the behavioral measures evaluated with an alpha of 0.05 and a power of 0.8 for the HET and HOM mice.** Longitudinal power analyses were run with Age as a factor (day for the P-2CST test), in addition to Genotype. In the analysis all the ages described in the Result Section were included when applicable.
(DOCX)

Appendix S1 **Detailed PhenoCube® statistics.**
(DOCX)

## References

[1] Menalled LB, Sison JD, Dragatsis I, Zeitlin S, Chesselet MF (2003) Time course of early motor and neuropathological anomalies in a knock-in mouse model of Huntington’s disease with 140 CAG repeats. J Comp Neurol 465: 11–26. 12926013 10.1002/cne.10776

[2] Roos RA, Hermans J, Vegter-van der Vlis M, van Ommen GJ, Bruyn GW (1993) Duration of illness in Huntington’s disease is not related to age at onset. J Neurol Neurosurg Psychiatry 56: 98–100. 8429330 10.1136/jnnp.56.1.98 PMC1014774

[3] Vonsattel JP, Myers RH, Stevens TJ, Ferrante RJ, Bird ED, et al (1985) Neuropathological classification of Huntington’s disease. J Neuropathol Exp Neurol 44: 559–577. 2932539 10.1097/00005072-198511000-00003

[4] The Huntington’s Disease Collaborative Research Group (1993) A novel gene containing a trinucleotide repeat that is expanded and unstable on Huntington’s disease chromosomes. The Huntington’s Disease Collaborative Research Group. Cell 72: 971–983. 8458085 10.1016/0092-8674(93)90585-e

[5] Gray M, Shirasaki DI, Cepeda C, Andre VM, Wilburn B, et al (2008) Full-length human mutant huntingtin with a stable polyglutamine repeat can elicit progressive and selective neuropathogenesis in BACHD mice. J Neurosci 28: 6182–6195. 18550760 10.1523/JNEUROSCI.0857-08.2008 PMC2630800

[6] Hickey MA, Chesselet MF (2003) The use of transgenic and knock-in mice to study Huntington’s disease. Cytogenet Genome Res 100: 276–286. 14526189 10.1159/000072863

[7] Menalled LB (2005) Knock-in mouse models of Huntington’s disease. NeuroRx 2: 465–470. 16389309 10.1602/neurorx.2.3.465 PMC1144489

[8] Heng MY, Duong DK, Albin RL, Tallaksen-Greene SJ, Hunter JM, et al (2010) Early autophagic response in a novel knock-in model of Huntington disease. Hum Mol Genet 19: 3702–3720. 20616151 10.1093/hmg/ddq285 PMC2935855

[9] White JK, Auerbach W, Duyao MP, Vonsattel JP, Gusella JF, et al (1997) Huntingtin is required for neurogenesis and is not impaired by the Huntington’s disease CAG expansion. Nat Genet 17: 404–410. 9398841 10.1038/ng1297-404

[10] Wheeler VC, White JK, Gutekunst CA, Vrbanac V, Weaver M, et al (2000) Long glutamine tracts cause nuclear localization of a novel form of huntingtin in medium spiny striatal neurons in HdhQ92 and HdhQ111 knock-in mice. Hum Mol Genet 9: 503–513. 10699173 10.1093/hmg/9.4.503

[11] Lin CH, Tallaksen-Greene S, Chien WM, Cearley JA, Jackson WS, et al (2001) Neurological abnormalities in a knock-in mouse model of Huntington’s disease. Hum Mol Genet 10: 137–144. 11152661 10.1093/hmg/10.2.137

[12] Heng MY, Tallaksen-Greene SJ, Detloff PJ, Albin RL (2007) Longitudinal evaluation of the Hdh(CAG)150 knock-in murine model of Huntington’s disease. J Neurosci 27: 8989–8998. 17715336 10.1523/JNEUROSCI.1830-07.2007 PMC6672210

[13] Rising AC, Xu J, Carlson A, Napoli VV, Denovan-Wright EM, et al (2011) Longitudinal behavioral, cross-sectional transcriptional and histopathological characterization of a knock-in mouse model of Huntington’s disease with 140 CAG repeats. Exp Neurol 228: 173–182. 21192926 10.1016/j.expneurol.2010.12.017 PMC3060971

[14] Hickey MA, Kosmalska A, Enayati J, Cohen R, Zeitlin S, et al (2008) Extensive early motor and non-motor behavioral deficits are followed by striatal neuronal loss in knock-in Huntington’s disease mice. Neuroscience 157: 280–295. 18805465 10.1016/j.neuroscience.2008.08.041 PMC2665298

[15] Brooks S, Higgs G, Jones L, Dunnett SB (2010) Longitudinal analysis of the behavioural phenotype in Hdh((CAG)150) Huntington’s disease knock-in mice. Brain Res Bull. 10.1016/j.brainresbull.2010.05.004 20457230

[16] Andrew SE, Goldberg YP, Kremer B, Telenius H, Theilmann J, et al (1993) The relationship between trinucleotide (CAG) repeat length and clinical features of Huntington’s disease. Nat Genet 4: 398–403. 8401589 10.1038/ng0893-398

[17] Lione LA, Carter RJ, Hunt MJ, Bates GP, Morton AJ, et al (1999) Selective discrimination learning impairments in mice expressing the human Huntington’s disease mutation. J Neurosci 19: 10428–10437. 10575040 10.1523/JNEUROSCI.19-23-10428.1999 PMC6782405

[18] Singer J (1998) Using SAS PROC MIXED to fit multilevel models,hierarchical models, and individuals growth models. Journal of Educational and Behavioral Statistics 24: 323–355.

[19] Benn CL, Fox H, Bates GP (2008) Optimisation of region-specific reference gene selection and relative gene expression analysis methods for pre-clinical trials of Huntington’s disease. Mol Neurodegener 3: 17. 18954449 10.1186/1750-1326-3-17 PMC2584034

[20] Sanberg PR, Fibiger HC, Mark RF (1981) Body weight and dietary factors in Huntington’s disease patients compared with matched controls. Med J Aust 1: 407–409. 6454826 10.5694/j.1326-5377.1981.tb135681.x

[21] Menalled LB, Chesselet MF (2002) Mouse models of Huntington’s disease. Trends Pharmacol Sci 23: 32–39. 11804649 10.1016/s0165-6147(00)01884-8

[22] von Horsten S, Schmitt I, Nguyen HP, Holzmann C, Schmidt T, et al (2003) Transgenic rat model of Huntington’s disease. Hum Mol Genet 12: 617–624. 12620967 10.1093/hmg/ddg075

[23] Lloret A, Dragileva E, Teed A, Espinola J, Fossale E, et al (2006) Genetic background modifies nuclear mutant huntingtin accumulation and HD CAG repeat instability in Huntington’s disease knock-in mice. Hum Mol Genet 15: 2015–2024. 16687439 10.1093/hmg/ddl125

[24] Woodman B, Butler R, Landles C, Lupton MK, Tse J, et al (2007) The Hdh(Q150/Q150) knock-in mouse model of HD and the R6/2 exon 1 model develop comparable and widespread molecular phenotypes. Brain Res Bull 72: 83–97. 17352931 10.1016/j.brainresbull.2006.11.004

[25] Fellows S, Schwarz M, Schaffrath C, Domges F, Noth J (1997) Disturbances of precision grip in Huntington’s disease. Neurosci Lett 226: 103–106. 9159500 10.1016/s0304-3940(97)00264-4

[26] Gordon AM, Quinn L, Reilmann R, Marder K (2000) Coordination of prehensile forces during precision grip in Huntington’s disease. Exp Neurol 163: 136–148. 10785452 10.1006/exnr.2000.7348

[27] Menalled L, El-Khodor BF, Patry M, Suarez-Farinas M, Orenstein SJ, et al (2009) Systematic behavioral evaluation of Huntington’s disease transgenic and knock-in mouse models. Neurobiol Dis 35: 319–336. 19464370 10.1016/j.nbd.2009.05.007 PMC2728344

[28] Hockly E, Cordery PM, Woodman B, Mahal A, van Dellen A, et al (2002) Environmental enrichment slows disease progression in R6/2 Huntington’s disease mice. Ann Neurol 51: 235–242. 11835380 10.1002/ana.10094

[29] Trembath MK, Horton ZA, Tippett L, Hogg V, Collins VR, et al (2010) A retrospective study of the impact of lifestyle on age at onset of Huntington disease. Mov Disord 25: 1444–1450. 20629137 10.1002/mds.23108

[30] van Dellen A, Blakemore C, Deacon R, York D, Hannan AJ (2000) Delaying the onset of Huntington’s in mice. Nature 404: 721–722. 10783874 10.1038/35008142

[31] Brooks SP, Betteridge H, Trueman RC, Jones L, Dunnett SB (2006) Selective extra-dimensional set shifting deficit in a knock-in mouse model of Huntington’s disease. Brain Res Bull 69: 452–457. 16624677 10.1016/j.brainresbull.2006.02.011

[32] Murphy C, Paterson N, Oakeshott S, He D, Alosio B, et al. (In preparation) Impairments in simple discrimination reversal and intra-dimensional shift in the BAC HD and z_Q175 mouse models of Huntington’s disease.

[33] Morton AJ, Skillings E, Bussey TJ, Saksida LM (2006) Measuring cognitive deficits in disabled mice using an automated interactive touchscreen system. Nat Methods 3: 767. 16990806 10.1038/nmeth1006-767

[34] Albin RL, Qin Y, Young AB, Penney JB, Chesselet MF (1991) Preproenkephalin messenger RNA-containing neurons in striatum of patients with symptomatic and presymptomatic Huntington’s disease: an in situ hybridization study. Ann Neurol 30: 542–549. 1838677 10.1002/ana.410300406

[35] Augood SJ, Faull RL, Emson PC (1997) Dopamine D1 and D2 receptor gene expression in the striatum in Huntington’s disease. Ann Neurol 42: 215–221. 9266732 10.1002/ana.410420213

[36] Bibb JA, Yan Z, Svenningsson P, Snyder GL, Pieribone VA, et al (2000) Severe deficiencies in dopamine signaling in presymptomatic Huntington’s disease mice. Proc Natl Acad Sci U S A 97: 6809–6814. 10829080 10.1073/pnas.120166397 PMC18747

[37] Chan EY, Luthi-Carter R, Strand A, Solano SM, Hanson SA, et al (2002) Increased huntingtin protein length reduces the number of polyglutamine-induced gene expression changes in mouse models of Huntington’s disease. Hum Mol Genet 11: 1939–1951. 12165556 10.1093/hmg/11.17.1939

[38] Denovan-Wright EM, Robertson HA (2000) Cannabinoid receptor messenger RNA levels decrease in a subset of neurons of the lateral striatum, cortex and hippocampus of transgenic Huntington’s disease mice. Neuroscience 98: 705–713. 10891614 10.1016/s0306-4522(00)00157-3

[39] Hebb AL, Robertson HA, Denovan-Wright EM (2004) Striatal phosphodiesterase mRNA and protein levels are reduced in Huntington’s disease transgenic mice prior to the onset of motor symptoms. Neuroscience 123: 967–981. 14751289 10.1016/j.neuroscience.2003.11.009

[40] Hu H, McCaw EA, Hebb AL, Gomez GT, Denovan-Wright EM (2004) Mutant huntingtin affects the rate of transcription of striatum-specific isoforms of phosphodiesterase 10A. Eur J Neurosci 20: 3351–3363. 15610167 10.1111/j.1460-9568.2004.03796.x

[41] Luthi-Carter R, Strand A, Peters NL, Solano SM, Hollingsworth ZR, et al (2000) Decreased expression of striatal signaling genes in a mouse model of Huntington’s disease. Hum Mol Genet 9: 1259–1271. 10814708 10.1093/hmg/9.9.1259

[42] McCaw EA, Hu H, Gomez GT, Hebb AL, Kelly ME, et al (2004) Structure, expression and regulation of the cannabinoid receptor gene (CB1) in Huntington’s disease transgenic mice. Eur J Biochem 271: 4909–4920. 15606779 10.1111/j.1432-1033.2004.04460.x

[43] Menalled L, Zanjani H, MacKenzie L, Koppel A, Carpenter E, et al (2000) Decrease in striatal enkephalin mRNA in mouse models of Huntington’s disease. Exp Neurol 162: 328–342. 10739639 10.1006/exnr.1999.7327

[44] Richfield EK, Maguire-Zeiss KA, Cox C, Gilmore J, Voorn P (1995) Reduced expression of preproenkephalin in striatal neurons from Huntington’s disease patients. Ann Neurol 37: 335–343. 7695232 10.1002/ana.410370309

[45] Squitieri F, Gellera C, Cannella M, Mariotti C, Cislaghi G, et al (2003) Homozygosity for CAG mutation in Huntington disease is associated with a more severe clinical course. Brain 126: 946–955. 12615650 10.1093/brain/awg077

