# Supplementary figures and images for: Comprehensive Behavioral and Molecular Characterization of a New Knock-In Mouse Model of Huntington’s Disease: zQ175
Source: PLoS One. 2012 Dec 20;7(12):e49838. doi: 10.1371/journal.pone.0049838 (PMC3527464; doi:10.1371/journal.pone.0049838)

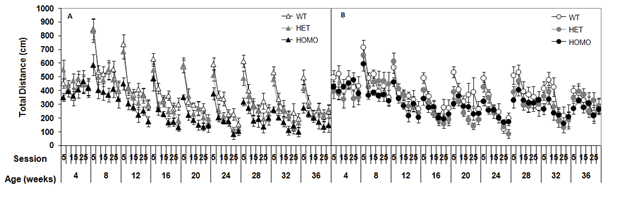

Supplement: Figure S1 — Total distance travelled (mean ± SEM) of wild type, heterozygous and homozygous zQ175 mice as a function of age for female (A) and male (B) mice during the light phase of the diurnal cycle. (DOCX) [file pone.0049838.s001.docx]

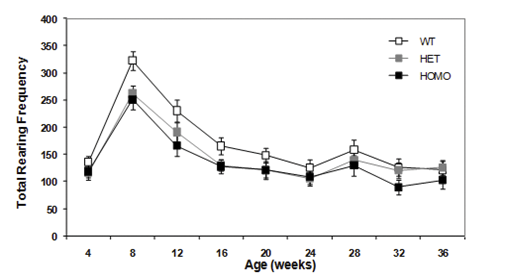

Supplement: Figure S2 — Rearing frequency (mean ± SEM) of wild type, heterozygous and homozygous mice as a function of age during the light phase of the diurnal cycle. (DOCX) [file pone.0049838.s002.docx]

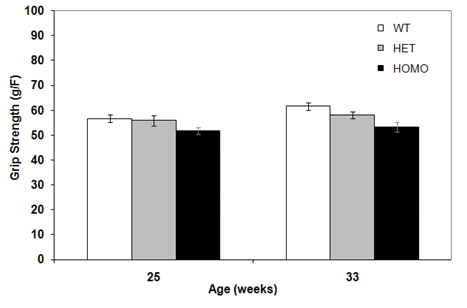

Supplement: Figure S3 — Average grip strength (mean ± SEM) of wild type, heterozygous and homozygous mice as a function of age during the dark phase of the diurnal cycle. (DOCX) [file pone.0049838.s003.docx]

**Table S2**


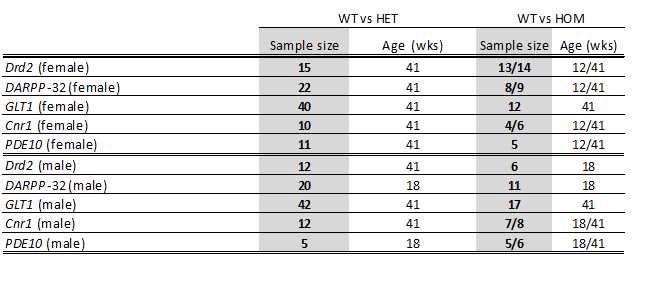

Supplement: Table S2 — Summarizes the sample size needed to detect a 50% effect in the transcripts evaluated with an alpha of 0.05 and a power of 0.8 for the HET and HOM mice. (DOCX) [file pone.0049838.s005.docx]
