# Supplementary material for: Comprehensive Behavioral and Molecular Characterization of a New Knock-In Mouse Model of Huntington’s Disease: zQ175
Source: PLoS One. 2012 Dec 20;7(12):e49838. doi: 10.1371/journal.pone.0049838 (PMC3527464; doi:10.1371/journal.pone.0049838)
Supplement: Table S1 — F and p value table for the significant main effects and interactions for the different gene markers examined for male and female mice. (DOCX) [file pone.0049838.s004.docx]

**Table S1**

|  | | Males | | Females | |
| --- | --- | --- | --- | --- | --- |
|  |  | F value | P value | F value | P value |
| ***D2dr*** | Genotype | F(2, 67) = 24.17 | < 0.0001 | F(2, 42) = 22.23 | < 0.0001 |
|  | Age | F(2, 67) = 26.88 | < 0.0001 | F(1,42) = 6.84 | < 0.05 |
|  | Genotype x age | F_(4,67)_=9.44 | < 0.0001 | F_(2,42)_=4.31 | < 0.05 |
| ***DARPP32*** | Genotype | F(2, 67) = 16.91 | < 0.0001 | F(2,42) = 26.65 | < 0.0001 |
|  | Age | F_(2,67)_=14.18 | < 0.0001 |  |  |
|  | Genotype x age | F_(4,67)_=6.77 | < 0.0001 |  |  |
| ***GLT1*** | Genotype | F(2, 67) =6.23 | p < 0.005 | F(2, 42) = 14.16 | < 0.0001 |
|  | Age | F_(2,67)_=5.36 | < 0.01 | F(1,42) = 12.60 | < 0.001 |
|  | Genotype x age |  |  | F_(2,42)_=3.60 | < 0.05 |
| **Cnr1** | Genotype | F(2, 67) = 13.27 | < 0.0001 | F(2, 42) = 35.87 | < 0.0001 |
|  | Age | F(2, 67) = 11.36 | < 0.05 | F(1, 42) = 9.47 | < 0.05 |
|  | Genotype x age | F_(4,67)_= 4.19 | < 0.005 | F_(4,67)_=4.80, | < 0.05 |
| **PDE10** | Genotype | F(2, 67) = 19.68 | < 0.0001 | F(2,42) = 43.06, | < 0.0001 |
|  | Age | F(2, 67) = 25.52 | < 0.0001 |  |  |
|  | Genotype x age | F_(4,67)_=7.72 | < 0.0001 |  |  |
