# Supplementary material for: Comprehensive Behavioral and Molecular Characterization of a New Knock-In Mouse Model of Huntington’s Disease: zQ175
Source: PLoS One. 2012 Dec 20;7(12):e49838. doi: 10.1371/journal.pone.0049838 (PMC3527464; doi:10.1371/journal.pone.0049838)
Supplement: Table S3 — Summarizes the sample size needed to detect a 50% effect in the behavioral measures evaluated with an alpha of 0.05 and a power of 0.8 for the HET and HOM mice. Longitudinal power analyses were run with Age as a factor (day for the P-2CST test), in addition to Genotype. In the analysis all the ages described in the Result Section were included when applicable. (DOCX) [file pone.0049838.s006.docx]

**Table S3**
