# Supplementary material for: Comprehensive Behavioral and Molecular Characterization of a New Knock-In Mouse Model of Huntington’s Disease: zQ175
Source: PLoS One. 2012 Dec 20;7(12):e49838. doi: 10.1371/journal.pone.0049838 (PMC3527464; doi:10.1371/journal.pone.0049838)
Supplement: Appendix S1 — Detailed PhenoCube® statistics. (DOCX) [file pone.0049838.s007.docx]

## Appendix SI: Detailed PhenoCube® statistics

PhenoCube – visit frequency. While there was a trend towards a four-way gender x genotype x test age x light/dark cycle interaction, F(3, 119) = 2.63, p < 0.06, there were no significant effects or interactions involving gender, F(1, 119) = 2.21, F(2, 119) = 1.54, larger remaining F(3, 119) = 1.08, all ps > 0.1. Follow-up analysis of the significant genotype x test age x day/night cycle interaction confirmed that there were significant effects of test age only during the night periods and then only in WT and zQ175 heterozygous mice, smaller F(1,119) = 14.07, ps < 0.001, with no significant differences in daytime behavior and no changes in the zQ175 homozygous animals in either day or night, largest F(1,119) = 1.65, ps > 0.2. There were highly significant differences between day and night performance in all groups of mice at both ages, smallest F(1,119) = 89.7, all ps < 0.0001, reflecting the clear activity cycling seen in these mice. Importantly, there were significant effects of genotype during the night periods at both test ages, smaller F(2,119) = 7.62, ps < 0.001, with no significant effects of genotype emerging in the daytime, Fs < 1, ps > 0.4.
